# Comparison of two commercial carbapenemase gene confirmatory assays in multiresistant *Enterobacteriaceae* and *Acinetobacter baumannii*-complex

**DOI:** 10.1371/journal.pone.0197839

**Published:** 2018-05-21

**Authors:** Stephan Rösner, Kevin Gehlweiler, Uta Küsters, Mathias Kolbert, Kirsten Hübner, Niels Pfennigwerth, Dietrich Mack

**Affiliations:** 1 Microbiology/Infectious Diseases, Bioscientia Institute for Medical Diagnostics, Ingelheim, Germany; 2 Department of Medical Microbiology, Ruhr-University Bochum, Bochum, Germany; Brigham and Women’s Hospital Channing Division of Network Medicine, UNITED STATES

## Abstract

Multidrug-resistant Gram-negative bacilli (MDR-GNB) producing carbapenemases are increasing at an alarming speed. Rapid confirmation of carbapenemase type will be an important diagnostic step in clinical microbiology laboratories not only to reduce the risk of transmissions but also for optimising antibiotic therapy in the future. We compared diagnostic reliability of two commercially available molecular assays (Check-Direct CPE vs. AID line probe assay) for detection and typing of carbapenemase genes in 80 well-characterized isolates of MDR-GNB. Respective strains were isolated in various clinical specimens at our clinical microbiology laboratory. The reference standard included confirmation of carbapenemase-production at the molecular level at the German National Reference Laboratory for Multidrug-resistant Gram-negative bacteria (Ruhr-University Bochum, Germany). 53 *Enterobacteriaceae* and 27 members of the *A*. *baumannii*-complex were used in this study. The tested assays appeared highly reliable to confirm carbapenemase-producing *Enterobacteriaceae* (CPE) with respective sensitivities of 97.7%, but are currently unsuitable for analysis of members of the *A*. *baumannii*-complex. Both assays are easy to perform and rapid tools for confirmation and typing of the most common carbapenemase genes in *Enterobacteriaceae*. Implementation should be possible for any clinical microbiology laboratory with Check-Direct CPE being easier to handle and having less technological requirements.

## Introduction

Multidrug-resistant Gram-negative bacilli (MDR-GNB) producing carbapenemases are increasing at an alarming speed and considered to be a significant threat to patient safety [1–3]. Carbapenemases belong to three molecular classes according to the Ambler classification: molecular class A (i.e. KPC types), molecular class B (i.e. VIM, IMP and NDM types), molecular class D (i.e. OXA-48-like enzymes). The NDM, OXA-48-like, KPC, IMP and VIM types are the most common global carbapenemases among carbapenemase-producing *Enterobacteriacae* (CPE) [1,4]. Among *A*. *baumannii* OXA-23 is the most prevalent carbapenemase in Europe [5,6].

Treatment of patients suffering from infections with carbapenemase-producing MDR-GNB is difficult as only a few alternative treatment options remain [7]. Being one of the last-line options, the consumption of polymyxins, particularly colistin, almost doubled in Europe between 2009 and 2013 [2]. Carbapenemases are capable to hydrolyze most β-lactams, including carbapenems, and most enzymes are not inhibited by clinically available β-lactamase inhibitors [1,3]. Avibactam, a non-β-lactam β-lactamase inhibitor, recently became commercially available and inhibits class A and partially class D carbapenemases. It is not active against class B carbapenemases [8]. Infections due to carbapenemase-producers show high mortality [9]. Thus, rapid confirmation of carbapenemase type will be an important diagnostic step in clinical microbiology laboratories not only to reduce the risk of transmissions but also for optimising antibiotic therapy for these difficult-to-treat bacteria in the future. A wide array of newer techniques for detection of carbapenemases has become available recently [4]. The goal of this study was to compare diagnostic reliability of two commercially available molecular assays for detection and typing of carbapenemase genes in cultured MDR-GNB-isolates.

## Material and methods

### Bacterial isolates

Between August 2012 and August 2016 146 MDR-GNB with decreased carbapenem susceptibility were isolated from various clinical specimens in our laboratory and carbapenemase-production was confirmed at the molecular level at the German National Reference Laboratory (NRL) for Multidrug-resistant Gram-negative bacteria (Ruhr-University Bochum, Germany). Copy strains were excluded (Table 1). Isolates were stored at -80 °C. Out of the 146 MDR-GNB-isolates with NRL-confirmed carbapenemase-status all carbapenemase-positive *Enterobacteriaceae* (n = 43, OXA-48 n = 15, KPC-2 n = 8, KPC-3 n = 2, NDM n = 9, VIM-1 n = 7, VIM-4 n = 1, IMP-14 n = 1) and 10 carbapenemase-negative *Enterobacteriaceae* were selected for analysis. One VIM-1-positive isolate was excluded, as on retesting it no longer showed decreased carbapenem susceptibility. All 27 members of the *A*. *baumannii*-complex (OXA-23 n = 21, OXA-72 n = 2, OXA-255 n = 1, GIM-1 n = 1, carbapenemase-negative n = 2) were subjected to analysis by both assays. After incubating overnight at 36 °C on blood agar (Beckton Dickinson, Heidelberg, Germany), a cell suspension (McFarland 0.5–1) was prepared using UltraPure^™^ DNase/RNase-Free Distilled Water (Thermo Fisher Scientific, Waltham, USA) and subjected to both tests.

**Table 1:** Strains, isolated in our laboratory and tested for carbapenemase production at the NRL Bochum between 08/2012 and 08/2016.

	Tested MDR-GNB (n)	Carbapenemase positive (n)
***Enterobacteriaceae***	118	44 (37.3%)
*E*. *coli*	26	8 (30.8%)
*Klebsiella spp*.^a^	41	23 (56.1%)
*E*. *cloacae*-complex	21	6 (28.6%)
*E*. *aerogenes*	16	0 (0.0%)
other *Enterobacteriaceae*	14	7 (50%)
***A*. *baumannii*-complex**	27	25 (92.6%)

^a^*Klebsiella spp*.: *K*. *pneumoniae* n = 36, *K*. *oxytoca* n = 3, *K*. *ornithinolytica* n = 1, *K*. *planticola* n = 1; *E*. *cloacae-*complex: *E*. *cloacae* n = 18, *E*. *kobei* n = 3; *A*. *baumannii*-complex: *A*. *baumannii* n = 25, *A*. *pittii* n = 2.

### AID line probe assay

The AID carbapenemase line probe assay RDB2290 (GenID^®^, Straßberg, Germany) allows the genetic detection of various carbapenemases by reverse hybridization (Table 2). The assay is able to discriminate between 13 carbapenemases (AIM, BIC, DIM, GIM, IMI, IMP, KPC, NDM, NMC-A, OXA-48, SIM, SPM, VIM). The report does not provide a specific variant of these carbapenemase gene families. The assay steps were carried out according to the user manual. The undiluted cell suspensions were subjected to automated DNA extraction by MagNAPure 96 (Roche Diagnostics, Mannheim, Germany). PCR was performed in a Labcycler (SensoQuest, Göttingen, Germany). For each reaction, 25 μl reaction mixture consisted of 15 μl Primer Nucleotide Mix (PN-Mix Carba), 2.5 μl 10x polymerase buffer (Qiagen, Hilden, Germany), 2 μl UltraPure^™^ DNase/RNase-Free Distilled Water (Thermo Fisher Scientific, Waltham, USA), 0.5 μl (= 2.5 units) HotStarTaq *Plus* DNA Polymerase (Qiagen, Hilden, Germany) and 5 μl sample DNA. Thermal cycling conditions were used as proposed by AID. Detection of the biotinylated amplicons was performed by reverse hybridization with the carbapenemase probes on the provided nitrocellulose strips. Results were interpreted per the AID instructions. The total time to perform AID line probe assay from cultural isolates to result is approximately 300 minutes.

**Table 2:** Detectable carbapenemases for each assay according to the respective user manual.

	AID line probe assay	Check-Direct CPE
**KPC**	KPC-1, 2, 3, 4, 5, 6, 7, 8, 9, 10	KPC-2, 3, 4, 5, 6, 7, 8, 9, 10, 11, 12, 13, 14, 15, 16, 17, 18, 19, 21, 22, 23, 24, 25
**OXA**	OXA-48, 199	OXA-48, 48b, 162, 163, 181, 204, 232, 244, 245, 370
**VIM**	VIM-1, 2, 4, 5, 8, 9, 10, 11, 12, 14, 15, 16, 17, 19, 20, 23, 24, 25, 26, 27, 28, 30, 31, 32, 33, 34, 35, 37, 38, 39, 40, 41, 42, 43, 44, 45, 46	VIM-1, 2, 3, 4, 5, 6, 8, 9, 10, 11, 12, 13, 14, 15, 16, 17, 18, 19, 20, 23, 24, 25, 26, 27, 28, 29, 30, 31, 32, 33, 34, 35,36, 37, 38, 39, 40, 41, 42, 43, 44, 45, 46, 47
**NDM**	NDM-1, 2, 3, 4, 5, 6	NDM-1, 2, 3, 4, 5, 6, 7, 8, 9, 10, 11, 12, 13, 14, 15, 16
**IMP**	IMP-1, 2, 4, 5, 6, 7, 8, 9, 10, 11, 13, 14, 15, 18, 19, 20, 21, 24, 25, 26, 28, 29, 30, 32, 33, 37, 38, 40, 41, 42, 43, 44, 45, 48, 49, 51	
**IMI**	IMI-1, 2, 3, 4, 7	
**Other**	AIM-1, BIC-1, DIM-1, GIM-1, NMC-A, SIM-1, SIM-2, SPM-1	

### Check-Direct CPE

Check-Direct CPE (Check-Points Health, Wageningen, The Netherlands) detects the presence of the carbapenemases KPC, NDM, VIM and OXA-48 by multiplex real-time PCR (Table 2). The assay discriminates between KPC, OXA-48, and NDM/VIM, it does not report a specific variant of the carbapenemase gene families. As NDM and VIM are detected by the same fluorochrome, it is not possible to differentiate between these two types of carbapenemases. According to the user manual, crude DNA was extracted from bacterial cell suspension by heating at 98 °C for 10 minutes followed by centrifugation at 21255 g for 2 minutes. PCR reactions were performed using a Rotor-Gene Q/Corbett Rotor-Gene 6000 (Qiagen, Hilden, Germany) according to the proposed Rotor-Gene Q program. Results were interpreted per the Check-Points instructions with the Rotor-Gene Q Software (Qiagen, Hilden, Germany). The total time to perform Check-Direct CPE from cultural isolate to result is approximately 150 minutes.

## Results

### Diagnostic reliability

Among *Enterobacteriaceae* OXA-48 was the most frequently detected carbapenemase. Among *A*. *baumannii*-complex OXA-23 was the predominant carbapenemase. Both assays missed IMP-14 with IMP-14 being in the proposed spectrum of AID line probe assay but not Check-Direct CPE (Table 3). ID and phenotype of the respective isolate (*Enterobacter cloacae*, MIC imipenem 1 μg/ml, MIC meropenem 4 μg/ml) were confirmed after analysing the organisms from fresh stock cultures.

**Table 3:** Tested carbapenemase genes and results for gold standard, AID line probe assay and Check-Direct CPE.

	Carbapenemase detected by gold standard (NRL Bochum)	n	Confirmed by AID line probe assay	Confirmed by Check-Direct CPE
***Enterobacteriaceae***	OXA-48	15	15	15
	KPC-2	8	8	8
	KPC-3	2	2	2
	NDM	9	9	9
	VIM-1	7	7	7
	VIM-4	1	1	1
	IMP-14	1	0	0
	None	10	10	10
***A*. *baumannii*-complex**	GIM-1	1	1	0
	OXA-23	21	0	0
	OXA-255	1	0	0
	OXA-72	2	0	0
	None	2	2	2

Both assays correctly confirmed 42/43 (97.7%) of the carbapenemase-producing *Enterobacteriaceae*-strains. One of twenty-five (4%) carbapenemase-positive members of *A*. *baumannii*-complex were detected by AID line probe assay, while Check-Direct CPE detected none (Table 3). Both tests showed no false-positive reactions, neither in carbapenemase-negative isolates, nor in carbapenemase-producing MDR-GNB (e.g. different than those detected by the reference analysis).

## Discussion

The main goal of the current study was to find a reliable method for confirmation and typing of carbapenemases in phenotypically suspicious cultured isolates in order to provide guidance for antibiotic therapy and to rapidly institute infection control measures for these difficult-to-treat bacteria. Thus, we compared the commercially available AID line probe assay with the Check-Direct CPE multiplex-PCR in well-characterized cultured MDR-GNB-isolates.

Our results show, that the tested assays are highly reliable to confirm CPE as respective sensitivities were 97.7%. Both assays failed to detect IMP-14 with IMP-14 being in the proposed spectrum of AID line probe assay but not Check-Direct CPE.

In line with current epidemiology both tests appeared unsuitable for analysis of members of the *A*. *baumannii*-complex. It appears highly desirable to augment these assays with the capability of detecting OXA-23, as this enzyme may also emerge in *Escherichia coli* [10,11]. However, no false-positive reactions occurred, neither in carbapenemase-negative isolates, nor in carbapenemase-producing MDR-GNB. As the number of carbapenemase-negative *A*. *baumannii*-complex strains was very low, the *A*. *baumannii* strains with carbapenemases not targeted by the assays are negative specificity controls for this line of bacteria.

Assuming that both assays are able to detect their predicted carbapenemase targets, we calculated expected sensitivities using the nationwide German data of the NRL Bochum of the year 2015 [6]. Expected sensitivities were high for both assays (data not shown) being higher for Check-Direct CPE as it possesses a broader spectrum among the most prevalent carbapenemases OXA-48-like, KPC, NDM, and VIM, respectively (Table 2), but this awaits further study.

A recently published study showed also excellent diagnostic reliability using AID line probe assay for cultured isolates. In contrast, direct molecular testing of urine samples revealed problems with specificity/positive predictive values as positive AID line probe assay-results could not be confirmed by culture methods [12]. This fact is also true for using Check-Direct CPE in primary specimens (e.g. perirectal swabs). In a published clinical study, in only 16% of the Check-Direct CPE-positive perirectal swabs a corresponding carbapenemase-producing organisms could be identified by culture methods. Thus, the positive predictive value (PPV) was only 21% [13]. A significant false-positive rate and low PPV are also described in a similar study evaluating Check-Direct CPE for direct analysis of rectal swabs [14].

In general, false-positive results seem to be appearing in many studies performing molecular carbapenemase testing in stool samples or rectal swabs [13,15,16]. Interpretation of such molecular results can be difficult as the epidemiological and clinical relevance is not known [15].

The major strength of our study was the utilization of well-characterized MDR-GNB-isolates that were analysed by the German NRL for Multidrug-resistant Gram-negative bacteria as reference standard. There, confirmation of carbapenemase-production was performed on a molecular level. By intentionally using cultured isolates our aim was to focus not primarily on the hygiene and contact isolation issue using the assays for screening of primary specimens, but on confirmation of carbapenemase-production in phenotypically suspicious isolates of a relevant clinical sample (e.g. blood culture, urine culture), i.e. reduced carbapenem susceptibility and/or positive modified hodge test. So, we suggest that a combination of culture plus susceptibility testing and molecular methods is the ideal workflow.

A limitation of this study is, that the number of tested carbapenemase-negative isolates of the *A*. *baumannii*-complex was low as prevalence of carbapenemase-production (92.6%) was high in these MDR-GNB (Table 1).

Summarizing, both assays are easy to perform and rapid tools ideally allowing same day confirmation and typing of the most common carbapenemase genes in *Enterobacteriaceae*. Implementation should be possible for any clinical microbiology laboratory. Check-Direct CPE seems to be easier to handle in regard to extraction method, technician time, turn-around-time and technological needs.

Among CPE, Check-Direct CPE showed comparable sensitivity. Check-Direct CPE is not capable to distinguish between the metallo-β-lactamases (class B) NDM and VIM because both are detected by the same fluorochrome. In regard to the goal concerning the current needs, differentiation between class A, B and D will be sufficient, as the newly introduced β-lactamase inhibitor avibactam shows no activity against class B and partial activity against class D [8]. Currently, we would prefer implementing Check-Direct CPE for confirmation of carbapenemase-producing *Enterobacteriaceae* in our laboratory. Continuous surveillance of carbapenemase epidemiology is required as the sensitivity of both assays may change unfavourably if some of the rarer carbapenemase-types become more prevalent.
